# Crustal thickness control on Sr/Y signatures of recent arc magmas: an Earth scale perspective

**DOI:** 10.1038/srep08115

**Published:** 2015-01-29

**Authors:** Massimo Chiaradia

**Affiliations:** 1 Section of Earth and Environmental Sciences, University of Geneva, Rue des Maraîchers 13, 1205 Geneva, Switzerland

## Abstract

Arc magmas originate in subduction zones as partial melts of the mantle, induced by aqueous fluids/melts liberated by the subducted slab. Subsequently, they rise through and evolve within the overriding plate crust. Aside from broadly similar features that distinguish them from magmas of other geodynamic settings (e.g., mid-ocean ridges, intraplate), arc magmas display variably high Sr/Y values. Elucidating the debated origin of high Sr/Y signatures in arc magmas, whether due to mantle-source, slab melting or intracrustal processes, is instrumental for models of crustal growth and ore genesis. Here, using a statistical treatment of >23000 whole rock geochemical data, I show that average Sr/Y values and degree of maturation (MgO depletion at peak Sr/Y values) of 19 out of 22 Pliocene-Quaternary arcs correlate positively with arc thickness. This suggests that crustal thickness exerts a first order control on the Sr/Y variability of arc magmas through the stabilization or destabilization of mineral phases that fractionate Sr (plagioclase) and Y (amphibole ± garnet). In fact, the stability of these mineral phases is function of the pressure at which magma evolves, which depends on crustal thickness. The data presented show also that high Sr/Y Pliocene-Quaternary intermediate-felsic arc rocks have a distinct origin from their Archean counterparts.

Despite the fact that arc magmas have broadly similar features, which distinguish them from magmas of other geodynamic settings (mid-ocean-ridges, intra-plate), the controls of source versus crustal processes on their chemical composition and evolution remain unclear, owing, among others, to the controversial meaning of some critical geochemical indices. For instance, the cause of variability of Sr/Y values and of the degree of Fe_2_O_3tot_ enrichment during magmatic evolution (so called tholeiitic versus calc-alkaline trend) is hotly debated^1^,^2^,^3^,^4^,^5^. Magmatic arc-related Phanerozoic rocks with high Sr/Y values (also known as adakites or adakite-like: SiO_2_ ≥ 56 wt.%, Y < 10 ppm, Sr > 300 ppm, Sr/Y > 20, ^87^Sr/^86^Sr < 0.704: ref. 3) have attracted increasing attention during the last two decades because: (i) they resemble geochemically to the Archean trondhjemite-tonalite-granite (TTG) suite and as such may be key to interpret processes of intermediate-felsic crust generation during the Archean and the Phanerozoic^6^,^7^,^8^,^9^, and (ii) they are associated with major porphyry-type deposits worldwide^10^,^11^,^12^,^13^,^14^,^15^, main suppliers of copper and precious metals to our economy^16^.

Adakites have been initially defined as partial melts of young subducted oceanic crust under P-T conditions where garnet is stable and plagioclase is not^17^,^18^, a view subsequently widely supported^1^,^19^,^20^,^21^,^22^. However, alternative explanations^2^,^3^,^4^ have proposed that adakite-like rocks can be produced in arcs where the crust is thick enough to stabilize amphibole ± garnet in the mid- to lower crust assemblage crystallizing from hydrous basaltic to andesitic magmas^23^,^24^,^25^,^26^,^27^,^28^,^29^ and in the residue of partial melts of mafic lower crust^27^,^30^,^31^,^32^,^33^. Elucidating the debated origin of high Sr/Y signatures in arc magmas is essential to develop a comprehensive model of arc magma genesis^34^, constrain models of crust formation^6^,^7^,^8^,^9^ and understand magmatic processes leading to the genesis of porphyry-type deposits^10^,^11^,^12^,^13^,^14^,^15^. If the high Sr/Y signatures of arc magmas are due to intracrustal processes a systematic control by crustal thickness on Sr/Y values should be expected, because crustal thickness is the main parameter controlling the development of adakite-like signatures in the intracrustal model, through stabilisation of magmatic (±residual) amphibole ± garnet and destabilization of plagioclase in a thick crust^23^,^24^,^30^,^31^,^32^.

## Results

This work presents and discusses >23000 whole rock geochemical data (Georoc database available at http://georoc.mpch-mainz.gwdg.de/georoc/Entry.html) from magmatic rocks of 22 Pliocene-Quaternary arcs of the Earth (Tables S1–S4 in the Supplementary Information and full dataset in the Supplementary Database), with the aim to see whether first order correlations exist between the typical adakitic index (Sr/Y) and crustal thickness. Following the approach of ref. 5, data, filtered to exclude altered samples, have been treated statistically by calculating the median values of Sr/Y for intervals equal or bigger than 0.5 wt.% MgO between 0 and 10 wt.% MgO (Tables S2–S4). Each ≥0.5 wt.% MgO interval contained at least 10 Sr/Y values for which the median value was calculated (Tables S2–S4). The use of the other typical adakitic index (La/Yb) is hindered because few geochemical analyses of the rocks reported in the Georoc database have coupled values of La and Yb, thus rendering less significant the statistical treatment of La/Yb. Because entire arc segments are compared, this study looks at whether crustal thickness exerts a first order control on the development of high Sr/Y signatures, by pooling together large numbers of magmatic rocks distributed along hundreds of km of arc segments. I have used average crustal thicknesses of arc segments determined by ref. 35, who also provides relative uncertainties with respect to the average value as an indication of the degree of variability of the crustal thickness along the arc segment (Table S1). Uncertainties are always much smaller than the average value of crustal thickness for each arc segment (Table S1), which justifies pooling together data within each arc segment. The advantage of performing statistical treatment of a large number of data within each arc segment is that “anomalies” will be outweighed by the bulk of the sample population: the latter may thus yield trends that are eventually controlled by large-scale geological variables (like crustal thickness). This does not exclude the occurrence, within each arc, of subordinate amounts of rocks (within the available database) having a different origin than that here proposed.

**Figure 1:**
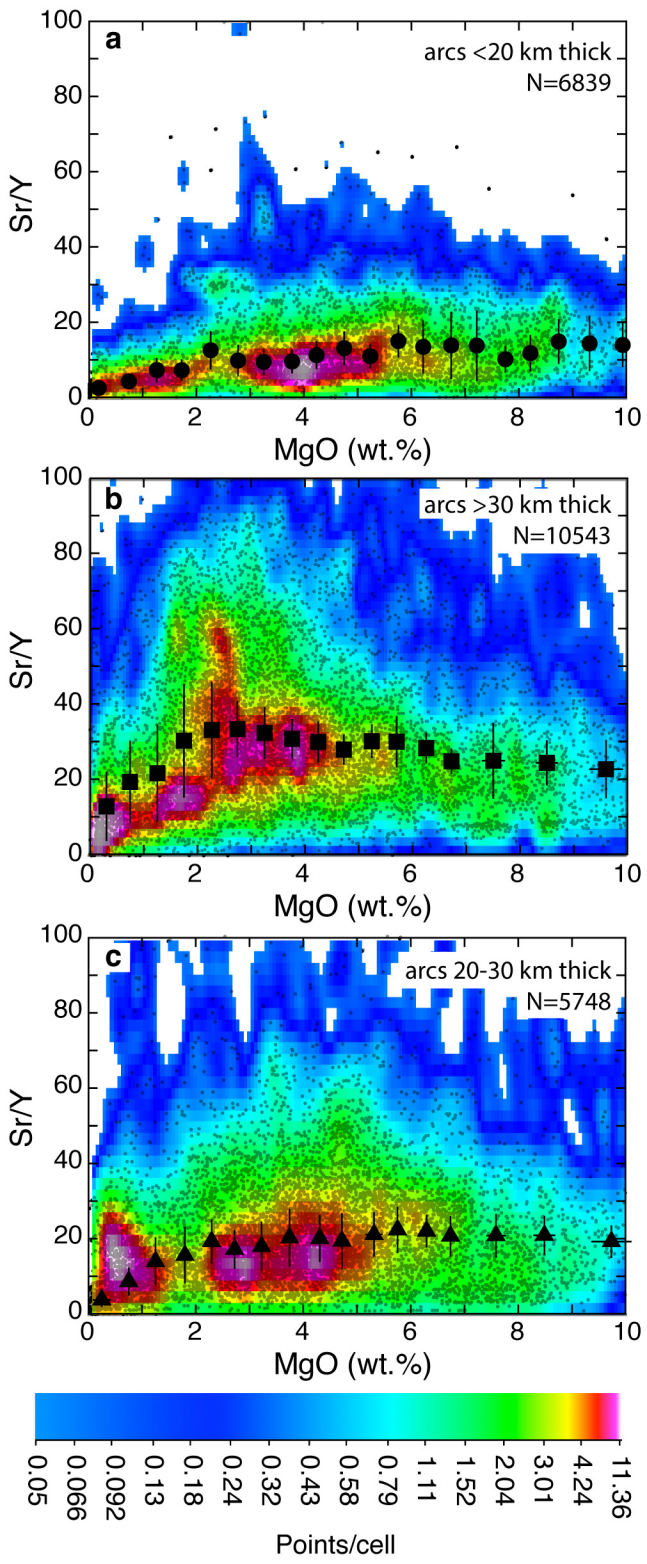
Sr/Y versus MgO plots of magmatic rocks from 22 Pliocene-Quaternary arcs. Small grey dots are individual rock analyses from the Georoc database with their density distribution (points/cell) indicated by color shadings. The number of analyses is indicated on the top right of each diagram. The large black symbols (circles 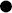, squares 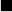, and triangles 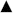) are averages of median MgO and Sr/Y values for intervals equal or bigger than 0.5 wt.% MgO for arcs <20 km, >30 km, and 20 to 30 km thick respectively. Bars are 1σ associated with the averages of median values (bars for MgO values are often smaller than symbol size).

Figure 1a shows that the averages of Sr/Y median values of all arcs <20 km thick (excluding the New Hebrides and Bismarck/New Britain arcs: see Table S2, Figures S1–S2 and below) remain constant (~15) between 10 and 6 wt.% MgO and then steadily decrease below 6 wt.% MgO. This suggests that magmatic differentiation occurs in the stability field of plagioclase, the main host of Sr in magmatic rocks especially during early differentiation processes (Tables S5–S6, Figure S7). In contrast, the averages of median Sr/Y values of arcs with Moho depth situated at >30 km steadily increase during magmatic differentiation (Figures 1b and S3–S4), starting from ~20 at 10 wt.% MgO and rising to 30–35 at intermediate-low MgO values (2–4 wt.%). This suggests that magmatic differentiation in these arcs occurs outside the stability field of plagioclase and within that of amphibole ± garnet (Y strongly partitioning into both amphibole and garnet during partial melting or magmatic fractionation processes: Tables S5–S6, Figure S7). Arcs with Moho depth ranging from 20 to 30 km display an Sr/Y versus MgO trend which lies between those of the two end-members above (Figures 1c and S5–S6; Tables S5–S6, Figure S7). Averages of median Sr/Y values of the three arc groups at ~10 wt.% MgO increase with crustal thickness (Figures 1 and S7), suggesting that the latter controls Sr/Y values also of relatively undifferentiated arc basalts (see also ref. 36 for a similar behaviour of Ce/Yb values in primitive arc basalts).

**Figure 2:**
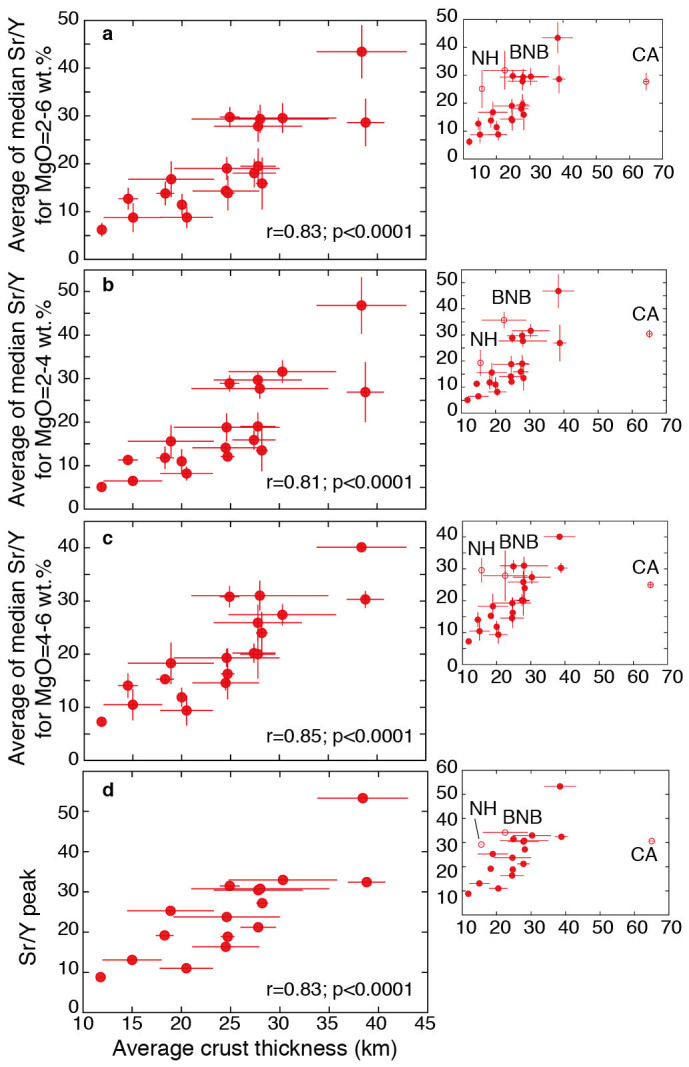
Correlations between crustal thickness and Sr/Y in 22 Pliocene-Quaternary arcs. (a). Plot of averages of median Sr/Y values corresponding to the 2–6 wt.% MgO interval versus average arc crust thickness (ref. 35); (b). Plot of averages of median Sr/Y values corresponding to the 2–4 wt.% MgO interval versus arc thickness; (c). Plot of averages of median Sr/Y values corresponding to the 4–6 wt.% MgO interval versus arc thickness; (d). Plot of the peak value of median Sr/Y values versus arc thickness (see Table S1 and Figures S2, S4, S6 in the Supplementary Information for details on how the peak values were calculated). Error bars for arc thicknesses are from ref. 35 (see Table S1). Error bars for Sr/Y values are standard deviations associated with the averages calculated from median values comprised within the considered MgO intervals (see Table S1). Peak Sr/Y values are from the best-fit exponential functions fitted through the median values and therefore have no standard deviation values associated (Figures S2, S4, S6). Abbreviations: CA = Central Andes; NH = New Hebrides; BNB = Bismarck-New Britain.

The systematic shift towards higher average Sr/Y values at intermediate MgO contents with increasing arc thickness (hinted by Figure 1) becomes evident in a bivariate plot of averages of median Sr/Y values between 2 and 6 wt.% MgO versus crustal thickness of the corresponding arc (Figure 2a). Excluding the New Hebrides and Bismarck/New Britain (see below) and the very thick Central Andes, all other 19 arcs plot along a statistically significant (r = 0.83; p < 0.0001) linear correlation trend (Figure 2a). Taking averages of median Sr/Y values for other MgO intervals (4–6 or 2–4 wt.%; Figures 2b–c) or peak median Sr/Y values (Figure 2d) of individual arcs does not change the goodness of the correlations for the 19 Quaternary arcs (0.81 ≤ r ≤ 0.85 and p < 0.0001 in all cases).

## Discussion

The linear correlations between Sr/Y values at intermediate MgO contents (Figures 2a–d) and crustal thickness of 19 out of 22 Pliocene-Quaternary arcs indicate that the great majority of recent arc magmatism owes, to a first order degree, its variable Sr/Y values to the control exerted by the upper plate thickness (see also ref. 37 for controls of crustal structure on the geochemistry of arc magmas). This makes it unlikely that source processes (slab melting or slab melt-mantle interactions) play a major role in the generation of high Sr/Y signatures in most recent arc magmas of the Earth.

The above correlations suggest that magmas of thicker arcs evolve at deeper average levels than those of thinner arcs: in fact, evolution at deeper levels stabilizes amphibole ± garnet versus plagioclase in the residual magma and in partial melts of mafic/intermediate protoliths^23^,^24^,^25^,^27^,^28^,^29^ resulting in higher Sr/Y values (Tables S5–S6, Figure S7). The thin New Hebrides and Bismarck/New Britain arcs (representing 4.7% of the total number of analytical data available, i.e. 1083 out of 23130 analyses) plot variably above the linear trends of Figures 2a–d indicating that they have average Sr/Y values somewhat higher than expected from the crustal thickness of those arcs. This supports the possibility that the high Sr/Y values of rocks from these arcs are due to source processes (e.g., slab melting^38^,^39^). Similarly also Miocene rocks of the Toyono Formation (in the <20 km thick Kurile arc) have adakitic signatures, possibly reflecting slab melting processes^40^.

**Figure 3:**
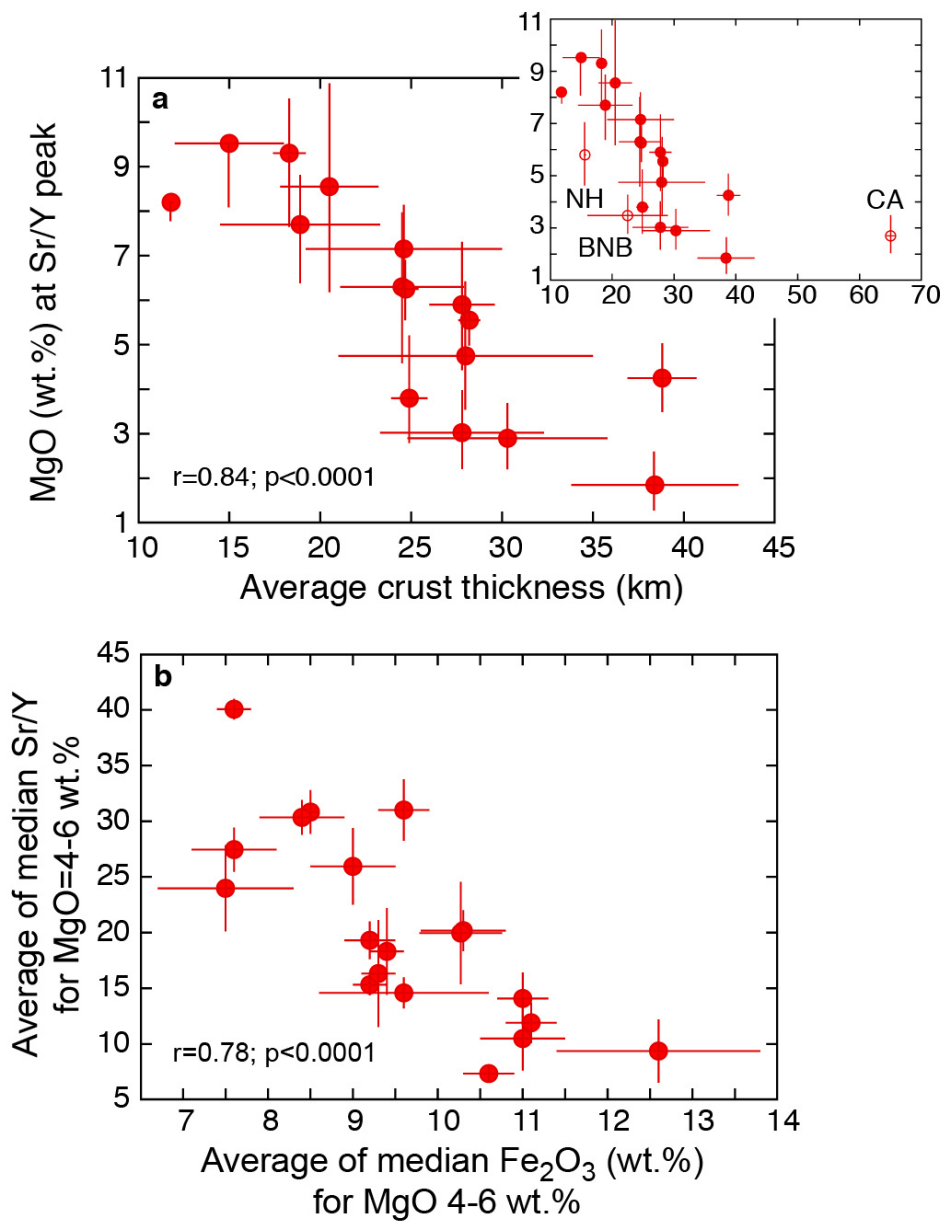
Correlations between crustal thickness, MgO, Sr/Y and Fe_2_O_3tot_ in 22 Pliocene-Quaternary arcs. (a). Plot of MgO values at which Sr/Y peaks occur (Figures S2, S4, S6) versus average crustal thickness (ref. 35). Horizontal bars are 1σ uncertainties of crustal thickness (ref. 35, Table S1). The extremities of the vertical bars associated with MgO represent the MgO values on either side of the Sr/Y peaks for corresponding Sr/Y values that are 5% lower than the peak value: this provides a measure of the narrowness of the peak and a control of the variability of the MgO content associated with the uncertain position of the Sr/Y peak (especially where the peak shape is flatter). For South Sandwich and Kermadec arcs the best-fit curves do not show peaks but monotonic decrease of Sr/Y with decreasing MgO (Figure S2), therefore there is no corresponding MgO value for the minus 5% upper side of the Sr/Y peak; (b). Plot of averages of median Sr/Y values corresponding to the 4–6 wt.% MgO interval of all arcs versus averages of median Fe_2_O_3tot_ values corresponding to the 4–6 wt.% MgO interval.

Thicker crust not only favours evolution of magmas at average deeper levels, but also leads to a more extensive magmatic differentiation at those levels. This is shown in Figure 3a where the MgO content at which the peak Sr/Y value occurs in each arc (Figures S2, S4, S6) is inversely correlated with arc thickness (r = 0.84; p < 0.0001): thus, the thicker is the arc the later in the magmatic differentiation process (i.e., at lower MgO contents) occurs the peak value of the Sr/Y ratio, which in turn is increasingly higher in thicker crust (Figures 2a–d). This is consistent with the model that increasing Sr/Y values are due to intracrustal evolution: where the crust is thicker magmas are likely to undergo a more extensive differentiation (resulting in progressively lower MgO contents) at higher average pressure conditions under which Y-bearing minerals (i.e., amphibole ± garnet) are stable and Sr-bearing plagioclase is not (resulting in higher Sr/Y values: Tables S5–S6, Figure S7).

It has been suggested that crustal thickness exerts a systematic control also on the development of the calc-alkaline versus the tholeiitic trend in recent arcs^5^. The evidence shown here for a systematic control by crustal thickness on Sr/Y values suggests that the more calc-alkaline the rocks are, the higher Sr/Y values they have. In fact, average Sr/Y values of magmatic arc rocks between 4–6 wt.% MgO correlate negatively with average Fe_2_O_3tot_ values for the same MgO interval (an expression of the tholeiitic degree of the magma, ref. 41: Figure 3b). The negative correlation between Sr/Y and such tholeiitic index suggests that magmas with the highest Sr/Y values are also the most calc-alkaline ones and implies that both the development of the calc-alkaline versus tholeiitic trend and high Sr/Y signatures are primarily the connected result of intracrustal processes. This is consistent with both high Sr/Y signatures and the calc-alkaline trend resulting from early fractionation of magnetite and amphibole and from suppression of plagioclase fractionation under high *p*H_2_O and *f*O_2 _conditions^41^,^42^,^43^,^44^,^45^. The latter conditions are apparently favoured by high-pressure intracrustal magmatic evolution^5^,^46^. The above results support also the idea that the association between high Sr/Y arc magmas and giant Cu-Au porphyry-type deposits is due to the intracrustal evolution of these magmas^12^,^13^,^14^,^15^ and not to processes at their source^47^,^48^.

**Figure 4:**
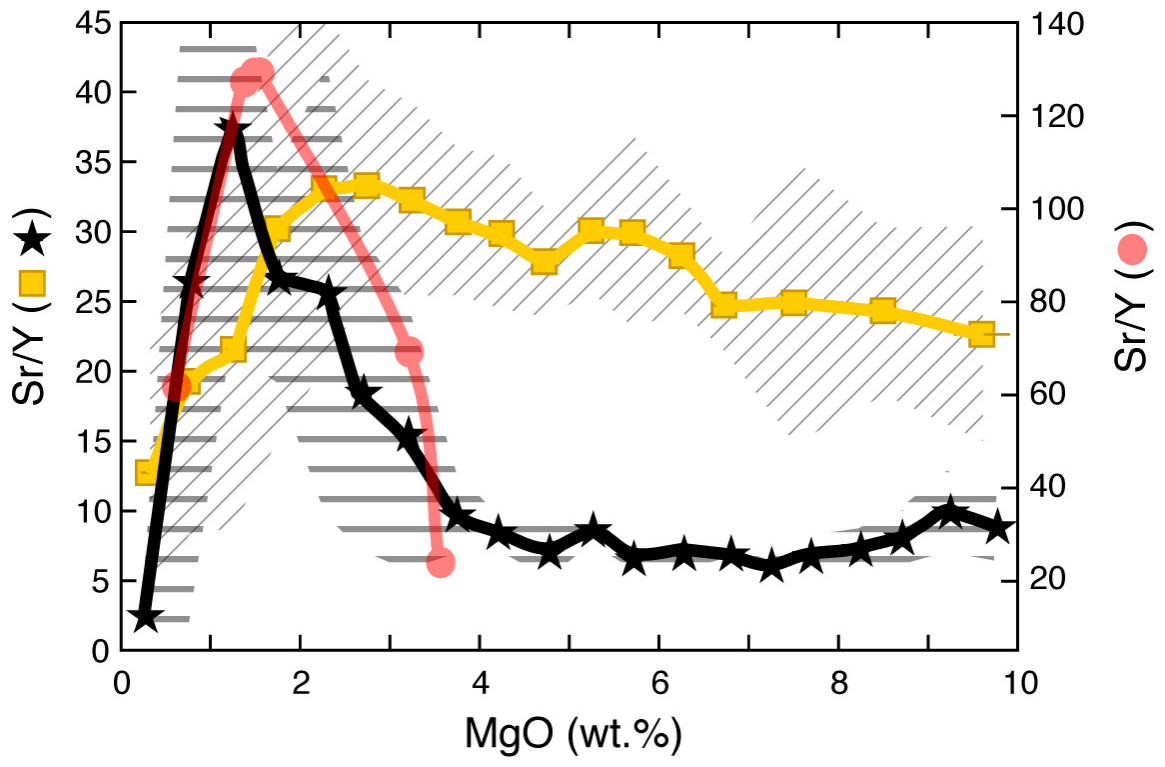
Comparison between Sr/Y versus MgO trends in Archean rocks and Pliocene-Quaternary arcs >30 km thick. Averages of median Sr/Y values of the arcs >30 km thick (yellow squares 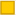) and of Archean greenstone belt rocks (black stars 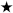: Baltic shield, Western Australia, Superior Province and Tanzania) versus MgO. Diagonal and horizontal lines areas represent the 1σ associated with the averages calculated for each group of rocks. Also reported are the compositions of experimental melts of mafic rocks at high-pressures (pale red circles 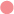) from ref. 56 (experiment AB1 at 3.8 GPa) and ref. 57 (all experiments at 1.5 GPa). The latter define an MgO-Sr/Y trend very similar to that of the Archean greenstone belt rocks.

Much debate exists on whether the Phanerozoic high Sr/Y magmatic rocks have been formed by processes similar to those that have produced the high Sr/Y signatures of TTG Archean magmatic rocks^9^,^49^,^50^,^51^ or not^22^,^52^,^53^,^54^. Figure 4 shows the averages of MgO versus Sr/Y median values from four Archean provinces (Western Australia, Superior Province, Baltic Shield, Tanzania craton: Table S7 and Figure S8) compared to the averages of MgO versus Sr/Y median values of Pliocene-Quaternary arcs >30 km thick. Whereas high Sr/Y values in thick Pliocene-Quaternary arcs result from a steady increase during magmatic differentiation of primitive basalts (10 wt.% MgO) with already elevated Sr/Y values (~20), Archean rocks display very low Sr/Y values (<10) throughout from 10 to 4 wt.% MgO after which Sr/Y values rise quickly forming a narrow peak (>35) between ~2.5 and ~0.5 wt.% MgO. Such a trend cannot be explained by a continuous fractionation of Y-bearing minerals in a thick crust, like for Pliocene-Quaternary arc magmas (Figure S7). It rather requires partial melting of mafic crust (either subducted oceanic crust or foundered lower crust: ref. 55) yielding high Sr/Y magmas with a narrow compositional interval (0.5–2.5 wt.% MgO) under pressure-temperature conditions for which amphibole and garnet are stable and plagioclase is not. This is consistent with experimental results of high-pressure partial melting of basalt^56^,^57^ that yield a trend in the Sr/Y versus MgO space very similar to that of the Archean rocks (Figure 4). Therefore, high Sr/Y Archean and Phanerozoic rocks have different origins reflecting two distinct processes of formation of juvenile felsic crustal rocks: lower- or infra-crustal partial melting of mafic crust during the Archean and intra-crustal fractionation of mafic magmas (with subordinate crustal melting and assimilation) during the Phanerozoic.

## Methods

The data plotted in Figures 1,2,3,4 and discussed in the text are from the Georoc database (http://georoc.mpch-mainz.gwdg.de/georoc/Entry.html). All samples that in the Georoc database were described as affected by any kind and degree of alteration were discarded. The remaining >23000 individual bulk rock analyses of 22 Pliocene-Quaternary arcs, developed upon continental and oceanic crust, and >3600 individual bulk analyses of Archean rocks have been statistically treated (Tables S1, S2, S7) and plotted in Figures 1,2,3,4. To reduce the bias induced by outliers and to extract information on general trends, data from each Pliocene-Quaternary arc and Archean province were statistically treated by calculating the median values of Sr/Y and MgO for subpopulations corresponding to intervals ≥~0.5 wt% MgO (see Tables S1, S2, S7 and Supplementary Dataset). Median values of Sr/Y comprised between 2–6, 2–4, and 4–6 wt.% MgO were averaged for each one of the 22 arcs (Tables S1 and S2) and corresponding uncertainties were calculated for the plots of Figs. 1,2,3. Also peak Sr/Y values were calculated from best-fit polynomial equations to the median values of each Pliocene-Quaternary arc and plotted against crustal thickness (Figures 2d, S2, S4, S6). In cases in which the best-fit curves yielded sinusoidal trends with more than one discernible peak, a peak value was not retained (Mariana, Tonga and Sulawesi arcs: Figures S2 and S4). MgO values corresponding to the Sr/Y peak values (Table S1) were used for the plot of Figure 3a. The extremities of the bars associated with these MgO values in Figure 3a represent the MgO values on either side of the Sr/Y peaks for corresponding Sr/Y values that are 5% less than the peak value: this gives a measure of the narrowness of the peak and provides a control of the variability of MgO associated with the uncertain position of the Sr/Y peak especially for flat peak shapes.

## Supplementary Material

Supplementary Information Supplementary Information

Supplementary Information Dataset 1
