# Supplementary material for: Crustal thickness control on Sr/Y signatures of recent arc magmas: an Earth scale perspective
Source: Sci Rep. 2015 Jan 29;5:8115. doi: 10.1038/srep08115 (PMC4309962; doi:10.1038/srep08115)
Supplement: Supplementary Information [file srep08115-s1.pdf]

Supplementary Information

Crustal thickness control on Sr/Y signatures of recent arc magmas: an Earth scale perspective

Massimo Chiaradia

Section of Earth and Environmental Sciences, University of Geneva, Rue des Maraîchers 13, 1205 Geneva, Switzerland

Materials and Methods

The data plotted in Figures 1-3 and discussed in the text are from the Georoc database (http://georoc.mpch-mainz.gwdg.de/georoc/Entry.html). All samples that in the Georoc database were described as affected by any kind and degree of alteration were discarded. The remaining >23000 individual bulk rock analyses of 22 Pliocene-Quaternary arcs, developed upon continental and oceanic crust, have been statistically treated (Tables S1-S2) and plotted in Figures 1-2.

Table S1: Parameters of the 22 arcs investigated in this study.

Arc	Crust thickness (km)[a]	1σ (km)	Sr/Y at 2-6 wt.% MgO[c]	1σ	Sr/Y at 2-4 wt.% MgO[c]	1σ	Sr/Y at 4-6 wt.% MgO[c]	1σ
South Sandwich	11.8	0.1	6.2*(6)*	1.4	5.1*(3)*	0.9	7.3*(3)*	0.7
Mariana	14.5	1.0	12.7*(8)*	2.3	11.3*(4)*	1.2	14.1*(4)*	2.3
Kermadec	15.0	3.0	8.8*(7)*	3.0	6.5*(3)*	1.1	10.5*(4)*	2.9
New Hebrides	15.6	0.2	25.2*(7)*	6.8	19.3*(3)*	5.0	29.6*(4)*	3.8
Kuriles	18.3	0.9	13.8*(7)*	2.5	11.8*(3)*	2.6	15.3*(4)*	0.9
Aleutians	18.9	4.4	16.8*(8)*	3.8	15.6*(4)*	3.7	18.3*(4)*	3.9
Tonga	20.0	-	11.5*(8)*	2.2	11.0*(4)*	2.8	11.9*(4)*	1.8
Izu-Bonin[b]	20.5	2.7	8.8*(8)*	2.2	8.2*(4)*	1.6	9.4*(4)*	2.8
Bismarck/NB[b]	22.5	6.5	31.8*(8)*	6.9	35.7*(4)*	3.1	27.9*(4)*	7.9
Ryukyu	24.5	3.4	14.3*(8)*	1.3	14.1*(4)*	1.3	14.6*(4)*	1.4
Kamchatka	24.6	5.4	19.0*(8)*	2.4	18.8*(4)*	3.2	19.3*(4)*	1.7
Lesser Antilles	24.7	0.7	13.9*(8)*	3.6	12.1*(4)*	0.7	16.3*(4)*	4.8
Aeolian	24.9	1.0	29.8*(8)*	2.1	28.9*(4)*	1.9	30.8*(4)*	2.0
Sulawesi	27.4	2.2	18.1*(4)*	3.0	15.9*(2)*	2.2	20.2*(2)*	1.8
Sunda	27.8	1.8	19.5*(8)*	3.7	19.0*(4)*	3.2	20.0*(4)*	4.6
Aegean	28.2	0.6	19.6*(8)*	5.5	13.5*(4)*	4.8	24.0*(4)*	3.9
Luzon[b]	27.8	4.5	27.8*(8)*	3.2	29.7*(4)*	1.7	25.9*(4)*	3.4
Central America[b]	28.0	7.0	29.4*(8)*	2.9	27.7*(4)*	2.3	31.0*(4)*	2.8
Mexico	30.3	5.5	29.6*(8)*	3.1	31.6*(4)*	2.6	27.4*(4)*	2.0
Ecuador	38.4	4.6	44.2*(8)*	6.3	46.8*(4)*	6.5	40.1*(4)*	0.9
Cascades	38.8	1.9	28.6*(8)*	5.0	26.9*(4)*	6.9	30.3*(4)*	1.6
Central Andes	65.0	0.7	27.7*(8)*	3.1	30.4*(4)*	1.6	25.0*(4)*	1.1

Table S1 (continued)

Arc	Sr/Y peak value [f]	MgO at peak Sr/Y [d]	Lower MgO at minus 5% of peak Sr/Y [d]	Upper MgO at minus 5% of peak Sr/Y [d]	MgO at peak Sr/Y [e]	Fe_2O_3 at 4-6 wt.% MgO [g]	1σ (wt.%)[f]	N of analyses [h]
South Sandwich	8.83	8.2	7.8	8.2	8.20	10.6	0.3	224
Mariana	i	i	i	i	8.73	11.0	0.3	506
Kermadec	13.11	9.52	8.11	9.52	9.52	11.0	0.5	406
New Hebrid.	29.2	5.8	4.67	7.01	5.74	11.2	0.9	467
Kuriles	19.2	9.3	7.67	10.55	7.25	9.2	0.2	526
Aleutians	25.3	7.7	6.4	8.84	6.71	9.4	0.2	1052
Tonga	i	i	i	i	6.28	11.1	0.3	1231
Izu-Bonin	11.03	8.55	6.2	10.9	5.76	12.6	1.2	1811
Bismarck/NB	34.2	3.48	2.82	4.24	3.25	9.8	0.3	616
Ryukyu	16.37	6.3	4.6	7.97	4.29	9.6	1.0	781
Kamchatka	23.77	7.15	6.06	8.15	6.76	9.2	0.3	975
Lesser Antilles	18.89	6.25	5.57	6.91	5.79	9.3	0.2	931
Aeolian	31.49	3.8	2.83	5.21	4.20	8.5	0.1	1064
Sulawesi	i	i	i	i	4.51	10.3	0.5	235
Sunda	21.22	5.9	4.45	7.32	5.74	10.3	0.5	969
Aegean	27.19	5.55	5	6.00	4.71	7.5	0.8	793
Luzon	30.44	3.025	2.22	3.99	2.26	9.0	0.5	888
Central America	30.78	4.75	3.55	6.44	4.74	9.6	0.3	2036
Mexico	32.98	2.9	2.22	3.69	2.29	7.6	0.5	2204
Ecuador	53.29	1.85	1.29	2.61	1.73	7.6	0.2	1435
Cascades	32.43	4.25	3.50	5.04	3.32	8.4	0.5	1696
Cent. Andes	30.64	2.7	2.09	3.45	2.73	8.2	0.5	2284

[a] From Zellmer (2008), except Tonga thickness, which is from Contreras et al. (2011), and Kermadec thickness, which is from Turner & Hawkesworth (1997). The Tonga crustal thickness here taken corresponds to the maximum crustal thickness of Contreras et al. (2011) because arc magmatism occurs in coincidence with the thickest part of the Tonga arc (Figure 9 in Contreras et al. (2011). Crustal thicknesses have been calculated by Zellmer (2008) using the global crustal model at 2x2 degrees, CRUST 2.0, administered by the US Geological Survey and the Institute for Geophysics and Planetary Physics at the University of California (Bassin et al. 2000), which is an updated version of CRUST 5.1, a global crustal model at 5x5 degrees Mooney et al. (1998). The model is based on seismic refraction data published up to 1995 and a detailed compilation of sediment thickness. The crustal thicknesses of Zellmer (2008) are within the ranges of crustal thicknesses reported in previous studies, with which they show good linear correlations (r=0.70 with respect to crustal thicknesses of Mantle and Collins (2008), and r=0.74 with respect to crustal thicknesses of Plank and Langmuir (1988).

[b] Attribution to a crust thickness type (Figures S1-S4) takes into account the 1σ uncertainty: for instance Izu-Bonin and Bismarck/New Britain (NB) have average crust thickness slightly above 20 km, but taking into account the 1σ uncertainty minimum values are largely <20 km and geochemical trends are more typical of arcs <20 km thick. The same applies to Luzon and Central America which are slightly <30 km thick but are attributed to the >30 km type due to the large 1σ uncertainty on crustal thickness. This subdivision is purely semantic and does not change the mathematical correlations of Figure 2.

[c] Averages and associated 1σ uncertainty are calculated from the median values reported in Tables S2-S5 for the intervals 2-6, 2-4, and 4-6 wt.% MgO. Numbers within brackets indicate the number of median values used to calculate the average and associated 1σ uncertainty.

[d] MgO value (wt.%) corresponding to the Sr/Y peak of the exponential best fit functions to the median values calculated for each arc (Figures S5-S7). The following two columns (MgO at minus 5% of peak Sr/Y value) represent the values of MgO calculated from the best fit functions at Sr/Y values that are 5% lower

than the peak value on either side of the peak. These values give an indication of the narrowness of the peak and therefore of the confidence on the corresponding MgO value. They are reported as bars in Figure 3a and fields in Figures S2, S4, S6. For South Sandwich and Kermadec arcs the best-fit curves do not show peaks but monotonic decrease of Sr/Y with decreasing MgO (Figure S2), therefore there is no corresponding MgO value for the minus 5% upper side of the Sr/Y peak.

[e] MgO value (wt.%) corresponding to the Sr/Y peak of the median values calculated within each arc (Figures S5-S7).

[f] Peak value of the Sr/Y median values (Tables S2-S5)

[g] From Chiaradia (2014)

[h] Number of analyses used from the Georoc database.

[i] Due to few median values and/or a sinusoidal trend the peak Sr/Y and corresponding MgO value cannot be reliably estimated (Figure S6).

References

Bassin, C., Laske, G. & Masters, T.G. The current limits of resolution for surface wave tomography in North America. *EOS Transactions of the AGU* **81**, F897 (2000).

Chiaradia, M. Copper enrichment in arc magmas controlled by overriding plate thickness. *Nature Geoscience* **7**, 43-46 (2014).

Contreras-Reyes, E. *et al.* Deep seismic structure of the Tonga subduction zone: Implications for mantle hydration, tectonic erosion, and arc magmatism. *J. Geophys. Res.* **116**, B10103 (2011).

Mantle, G.W. & Collins, W.J. Quantifying crustal thickness variations in evolving orogens: Correlation between arc basalt composition and Moho depth. *Geology* **36**, 87-90 (2008).

Mooney, W.D., Laske, G. & Masters, T.G. Crust 5.1: a global crustal model at 5x5 degrees. *J. Geophys. Res.* **103**, 727–747 (1998).

Plank, T. & Langmuir, C.H. An evaluation of the global variations in major element chemistry of arc basalts. *Earth Planet. Sci. Lett.* **90**, 349-370 (1988).

Turner, S. & Hawkesworth, C. Constraints on flux rates and mantle dynamics beneath island arcs from Tonga–Kermadec lava geochemistry. *Nature* **38**, 568-573 (1997).

Zellmer, G. Some first-order observations on magma transfer from mantle wedge to upper crust at volcanic arcs. *Geol. Soc. London Spec. Pub.* **304**, 15-31 (2008).

Table S2: Summary of median values of MgO (wt.%) and Sr/Y for arcs in thin crust (<20 km) calculated for the MgO intervals indicated. N = number of points within each interval.

Arc	MgO	Sr/Y	Interval	N	Arc	MgO	Sr/Y	Interval	N
Aleutians	0.18	1.52	0-0.5	24	Izu Bonin	7.76	11.13	7.5-8	74
Aleutians	0.69	4.58	0.5-1	36	Izu Bonin	8.19	11.00	8-8.5	49
Aleutians	1.24	6.56	1-1.5	23	Izu Bonin	8.72	11.23	8.5-9	60
Aleutians	1.85	7.97	1.5-2	52	Izu Bonin	9.26	9.22	9-9.5	37
Aleutians	2.29	21.06	2-2.5	65	Izu Bonin	9.88	12.17	9.5-10.2	39
Aleutians	2.76	12.83	2.5-3	86	Kermadec	0.89	3.24	0-1	12
Aleutians	3.30	15.05	3-3.5	111	Kermadec	1.31	4.00	1-1.5	72
Aleutians	3.76	13.56	3.5-4	108	Kermadec	1.69	4.42	1.5-2	42
Aleutians	4.23	13.81	4-4.5	120	Kermadec	2.90	5.17	2-3	20
Aleutians	4.72	20.41	4.5-5	70	Kermadec	3.26	7.12	3-3.5	40
Aleutians	5.29	23.75	5-5.5	58	Kermadec	3.75	7.20	3.5-4	25
Aleutians	5.76	20.69	5.5-6	50	Kermadec	4.23	8.55	4-4.5	30
Aleutians	6.15	26.43	6-6.5	41	Kermadec	4.74	9.97	4.5-5	57
Aleutians	6.71	28.30	6.5-7	20	Kermadec	5.17	8.73	5-5.5	19
Aleutians	7.27	27.91	7-7.5	19	Kermadec	5.78	14.66	5.5-6.5	29
Aleutians	8.14	20.09	7.5-8.5	31	Kermadec	7.03	9.88	6.5-7.5	31
Aleutians	8.77	21.20	8.5-9	21	Kermadec	7.84	11.25	7.5-8.3	14
Aleutians	9.25	25.03	9-9.5	18	Kermadec	9.52	13.63	8.3-10.5	15
Aleutians	9.93	20.48	9.5-10.1	19	Kuriles	0.04	2.04	0-0.5	67
Bismarck/NB	0.17	2.66	0-0.5	32	Kuriles	0.73	5.91	0.5-1	18
Bismarck/NB	0.73	7.72	0.5-1	22	Kuriles	1.18	8.90	1-1.5	14
Bismarck/NB	1.35	15.43	1-1.5	37	Kuriles	2.31	13.38	1.5-2.5	41
Bismarck/NB	1.80	12.00	1.5-2	46	Kuriles	3.05	8.78	2.5-3.5	40
Bismarck/NB	2.29	35.51	2-2.5	44	Kuriles	3.79	13.22	3.5-4	24
Bismarck/NB	2.80	35.00	2.5-3	25	Kuriles	4.26	16.45	4-4.5	27
Bismarck/NB	3.25	39.84	3-3.5	45	Kuriles	4.84	15.68	4.5-5	42
Bismarck/NB	3.80	32.31	3.5-4	33	Kuriles	5.26	14.53	5-5.5	40
Bismarck/NB	4.26	19.20	4-4.5	31	Kuriles	5.73	14.57	5.5-6	41
Bismarck/NB	4.76	32.50	4.5-5	42	Kuriles	6.22	13.86	6-6.5	36
Bismarck/NB	5.30	23.75	5-5.5	31	Kuriles	6.69	21.45	6.5-7	19
Bismarck/NB	5.80	36.33	5.5-6	43	Kuriles	7.25	22.96	7-7.5	19
Bismarck/NB	6.26	30.37	6-6.5	34	Kuriles	7.77	15.10	7.5-8	23
Bismarck/NB	6.77	15.86	6.5-7	44	Kuriles	8.30	14.59	8-8.5	30
Bismarck/NB	7.33	20.45	7-7.5	25	Kuriles	8.62	20.83	8.5-9	21
Bismarck/NB	7.72	11.96	7.5-8	19	Kuriles	9.97	19.39	9-10.75	12
Bismarck/NB	8.24	29.29	8-8.5	19	Marianas	0.19	3.74	0-1	14
Bismarck/NB	9.40	21.58	8.5-10.9	35	Marianas	1.57	7.49	1-1.6	16
Izu Bonin	0.33	2.96	0-0.5	79	Marianas	1.92	7.89	1.6-2	16
Izu Bonin	0.81	4.29	0.5-1	80	Marianas	2.17	9.53	2-2.5	43
Izu Bonin	1.17	4.94	1-1.5	70	Marianas	2.73	12.02	2.5-3	18
Izu Bonin	1.75	8.48	1.5-2	46	Marianas	3.27	11.97	3-3.5	29
Izu Bonin	2.30	9.66	2-2.5	34	Marianas	3.87	11.74	3.5-4	34
Izu Bonin	2.77	9.53	2.5-3	49	Marianas	4.31	12.00	4-4.5	41
Izu Bonin	3.23	7.07	3-3.5	67	Marianas	4.70	12.60	4.5-5	48
Izu Bonin	3.87	6.65	3.5-4	229	Marianas	5.25	14.55	5-5.5	37
Izu Bonin	4.17	6.98	4-4.5	174	Marianas	5.68	17.17	5.5-6	35
Izu Bonin	4.75	7.74	4.5-5	121	Marianas	6.20	7.00	6-6.5	31
Izu Bonin	5.24	9.34	5-5.5	144	Marianas	6.71	8.85	6.5-7	31
Izu Bonin	5.76	13.36	5.5-6	115	Marianas	7.26	6.41	7-7.5	31
Izu Bonin	6.24	12.24	6-6.5	101	Marianas	7.66	7.37	7.5-8	18
Izu Bonin	6.77	10.33	6.5-7	98	Marianas	8.21	9.14	8-8.5	22
Izu Bonin	7.24	9.84	7-7.5	86	Marianas	8.73	13.20	8.5-9.3	19

Table S2 (continued)

Arc	MgO	Sr/Y	Interval	N
New Hebrides	1.08	6.58	0.5-1.5	22
New Hebrides	1.80	12.17	1.5-2	17
New Hebrides	2.34	13.53	2-2.5	23
New Hebrides	2.77	21.32	2.5-3	17
New Hebrides	3.75	22.93	3-4	48
New Hebrides	4.31	25.31	4-4.5	47
New Hebrides	4.71	29.29	4.5-5	62
New Hebrides	5.24	29.19	5-5.5	31
New Hebrides	5.74	34.59	5.5-6	25
New Hebrides	6.29	26.17	6-6.5	27
New Hebrides	6.75	24.04	6.5-7	26
New Hebrides	7.25	24.17	7-7.5	19
New Hebrides	7.70	31.67	7.5-8	16
New Hebrides	8.30	23.01	8-8.5	15
New Hebrides	8.66	24.40	8.5-9	16
New Hebrides	9.64	21.04	9-10	22
S. Sandwich	1.54	2.84	0.8-2	21
S. Sandwich	2.80	4.50	2-3	15
S. Sandwich	3.31	4.71	3-3.5	25
S. Sandwich	3.68	6.08	3.5-4	25
S. Sandwich	4.20	7.28	4-4.6	22
S. Sandwich	5.31	6.65	4.6-5.5	37
S. Sandwich	5.82	8.07	5.5-6	22
S. Sandwich	6.22	7.40	6-6.5	23
S. Sandwich	6.77	8.27	6.5-7	19
S. Sandwich	8.20	8.79	7-10.9	15
Tonga	0.60	3.34	0-1	36
Tonga	1.42	12.06	1-1.5	51
Tonga	1.77	11.67	1.5-2	36
Tonga	2.26	9.13	2-2.5	44
Tonga	2.69	14.77	2.5-3	52
Tonga	3.33	11.38	3-3.5	81
Tonga	3.73	8.74	3.5-4	83
Tonga	4.26	13.82	4-4.5	135
Tonga	4.75	12.16	4.5-5	86
Tonga	5.17	12.17	5-5.5	82
Tonga	5.77	9.42	5.5-6	86
Tonga	6.28	13.65	6-6.5	61
Tonga	6.79	5.92	6.5-7	87
Tonga	7.24	5.67	7-7.5	70
Tonga	7.71	5.95	7.5-8	63
Tonga	8.24	7.11	8-8.5	59
Tonga	8.86	7.91	8.5-9	34
Tonga	9.22	9.67	9-9.5	31
Tonga	9.83	8.61	9.5-10.4	21

Table S3: Summary of median values of MgO and Sr/Y for arcs in intermediate crust (20-30 km) calculated for the MgO intervals indicated. N = number of points within each interval.

Arc	MgO	Sr/Y	Interval	N	Arc	MgO	Sr/Y	Interval	N
Aegean	0.49	4.83	0-1	167	Less. Antilles	5.79	21.87	5.5-6	22
Aegean	1.40	9.27	1-2	164	Less. Antilles	6.45	19.28	6-7	26
Aegean	2.68	13.49	2-3	120	Less. Antilles	7.50	15.00	7-8	31
Aegean	3.62	18.04	3-4	89	Less. Antilles	9.43	14.94	8-11.7	32
Aegean	4.43	21.00	4-5	141	Ryukyu	0.35	4.32	0-0.5	28
Aegean	5.48	28.00	5-6	68	Ryukyu	0.77	6.64	0.5-1	73
Aegean	6.78	14.24	6-10.45	42	Ryukyu	1.16	8.07	1-1.5	112
Aeolian	0.15	0.48	0-0.5	59	Ryukyu	1.77	12.97	1.5-2	43
Aeolian	0.85	9.23	0.5-1	45	Ryukyu	2.32	15.35	2-2.5	53
Aeolian	1.26	17.89	1-1.5	31	Ryukyu	2.71	13.14	2.5-3	58
Aeolian	1.80	29.00	1.5-2	43	Ryukyu	3.21	12.78	3-3.5	54
Aeolian	2.19	28.40	2-2.5	59	Ryukyu	3.81	14.93	3.5-4	66
Aeolian	2.80	26.59	2.5-3	69	Ryukyu	4.29	16.13	4-4.5	65
Aeolian	3.21	27.92	3-3.5	96	Ryukyu	4.73	15.04	4.5-5	55
Aeolian	3.72	31.47	3.5-4	128	Ryukyu	5.23	14.38	5-5.5	48
Aeolian	4.20	32.62	4-4.5	91	Ryukyu	5.70	12.80	5.5-6	31
Aeolian	4.76	32.27	4.5-5	82	Ryukyu	6.24	16.50	6-6.5	33
Aeolian	5.20	29.95	5-5.5	75	Ryukyu	6.88	17.38	6.5-7.5	30
Aeolian	5.79	28.46	5.5-6	57	Ryukyu	8.41	16.10	7.5-10.5	30
Aeolian	6.25	27.93	6-6.5	100	Sulawesi	0.63	4.98	0-1	24
Aeolian	6.69	28.60	6.5-7	73	Sulawesi	1.29	25.92	1-2	31
Aeolian	7.65	29.90	7-9.9	51	Sulawesi	2.56	17.52	2-3	39
Kamchatka	0.09	4.83	0-0.5	59	Sulawesi	3.54	14.35	3-4	41
Kamchatka	0.74	11.03	0.5-1	36	Sulawesi	4.51	21.47	4-5	29
Kamchatka	1.26	14.16	1-1.5	28	Sulawesi	5.52	18.88	5-6	29
Kamchatka	1.80	11.70	1.5-2	48	Sulawesi	6.66	17.59	6-7.5	21
Kamchatka	2.25	18.62	2-2.5	33	Sulawesi	8.41	28.99	7.5-11.7	21
Kamchatka	2.81	16.58	2.5-3	56	Sunda	0.18	4.11	0-0.5	55
Kamchatka	3.23	16.50	3-3.5	49	Sunda	0.78	5.44	0.5-1	32
Kamchatka	3.78	23.32	3.5-4	83	Sunda	1.16	8.07	1-1.5	37
Kamchatka	4.29	18.74	4-4.5	64	Sunda	1.75	12.15	1.5-2	43
Kamchatka	4.75	18.27	4.5-5	89	Sunda	2.36	20.69	2-2.5	72
Kamchatka	5.21	18.38	5-5.5	80	Sunda	2.79	20.57	2.5-3	103
Kamchatka	5.76	21.84	5.5-6	49	Sunda	3.25	20.54	3-3.5	111
Kamchatka	6.28	23.28	6-6.5	57	Sunda	3.78	14.30	3.5-4	112
Kamchatka	6.76	25.85	6.5-7	47	Sunda	4.20	16.99	4-4.5	93
Kamchatka	7.27	24.65	7-7.5	40	Sunda	4.68	17.51	4.5-5	62
Kamchatka	7.78	21.93	7.5-8	37	Sunda	5.22	18.54	5-5.5	48
Kamchatka	8.21	20.35	8-8.5	41	Sunda	5.74	26.79	5.5-6	54
Kamchatka	8.67	22.10	8.5-9	24	Sunda	6.25	23.15	6-6.5	38
Kamchatka	9.30	20.80	9-9.5	23	Sunda	6.63	20.00	6.5-7	23
Kamchatka	9.97	22.57	9.5-11	32	Sunda	7.67	15.75	7-8	26
Less. Antilles	0.77	14.58	0-1	25	Sunda	9.94	14.34	8-10.8	18
Less. Antilles	1.24	14.14	1-1.5	21					
Less. Antilles	1.88	12.12	1.5-2	25					
Less. Antilles	2.36	13.15	2-2.5	85					
Less. Antilles	2.80	11.71	2.5-3	183					
Less. Antilles	3.23	11.98	3-3.5	138					
Less. Antilles	3.77	11.48	3.5-4	119					
Less. Antilles	4.22	13.33	4-4.5	121					
Less. Antilles	4.68	13.77	4.5-5	50					
Less. Antilles	5.30	14.08	5-5.5	30					

Table S4: Summary of median values of MgO and Sr/Y for arcs in thick crust (>30 km) calculated for the MgO intervals indicated. N=number of points within each interval.

Arc	MgO	Sr/Y	Interval	N	Arc	MgO	Sr/Y	Interval	N
Cascades	0.37	10.39	0-0.5	78	Cent. Andes	8.66	27.75	8-9.45	24
Cascades	0.71	13.38	0.5-1	77	Ecuador	0.31	27.69	0-0.5	46
Cascades	1.35	14.23	1-1.5	94	Ecuador	0.76	39.22	0.5-1	21
Cascades	1.75	15.89	1.5-2	128	Ecuador	1.33	46.98	1-1.5	49
Cascades	2.20	17.23	2-2.5	95	Ecuador	1.73	57.81	1.5-2	159
Cascades	2.76	26.85	2.5-3	71	Ecuador	2.28	56.02	2-2.5	232
Cascades	3.32	31.30	3-3.5	121	Ecuador	2.74	47.94	2.5-3	235
Cascades	3.73	32.27	3.5-4	114	Ecuador	3.27	45.91	3-3.5	173
Cascades	4.23	32.53	4-4.5	88	Ecuador	3.75	40.24	3.5-4	170
Cascades	4.73	30.06	4.5-5	52	Ecuador	4.25	39.60	4-4.5	114
Cascades	5.31	28.83	5-5.5	111	Ecuador	4.73	40.31	4.5-5	114
Cascades	5.70	29.95	5.5-6	71	Ecuador	5.26	37.92	5-5.5	55
Cascades	6.27	26.79	6-6.5	50	Ecuador	5.65	41.72	5.5-6	21
Cascades	6.79	22.52	6.5-7	58	Ecuador	6.33	34.48	6-7	25
Cascades	7.30	9.00	7-7.5	78	Ecuador	7.87	35.00	7-11	21
Cascades	7.74	19.76	7.5-8	84	Luzon	0.40	8.80	0-0.6	28
Cascades	8.32	24.46	8-8.5	90	Luzon	0.88	20.98	0.6-1	67
Cascades	8.70	12.32	8.5-9	84	Luzon	1.24	11.24	1-1.5	89
Cascades	9.25	12.40	9-9.5	46	Luzon	1.75	32.28	1.5-2	59
Cascades	9.78	13.13	9.5-10.2	65	Luzon	2.26	32.08	2-2.5	104
Cent. America	0.35	19.39	0-0.5	348	Luzon	2.75	29.96	2.5-3	102
Cent. America	0.70	17.73	0.5-1	396	Luzon	3.22	28.55	3-3.5	79
Cent. America	1.12	18.36	1-1.5	107	Luzon	3.79	28.40	3.5-4	73
Cent. America	1.71	18.23	1.5-2	46	Luzon	4.26	25.80	4-4.5	65
Cent. America	2.30	28.18	2-2.5	99	Luzon	4.69	25.91	4.5-5	33
Cent. America	2.73	30.33	2.5-3	100	Luzon	5.24	30.23	5-5.5	29
Cent. America	3.23	27.56	3-3.5	108	Luzon	5.71	21.82	5.5-6	23
Cent. America	3.78	24.75	3.5-4	131	Luzon	6.32	23.17	6-6.5	23
Cent. America	4.22	27.09	4-4.5	146	Luzon	6.78	22.12	6.5-7	37
Cent. America	4.74	33.56	4.5-5	195	Luzon	7.32	19.53	7-8	36
Cent. America	5.21	31.59	5-5.5	127	Luzon	8.65	26.16	8-9	16
Cent. America	5.66	31.76	5.5-6	60	Luzon	9.99	26.27	9-11	19
Cent. America	6.22	31.59	6-6.5	41	Mexico	0.24	2.43	0-0.5	150
Cent. America	6.83	27.85	6.5-7	32	Mexico	0.69	6.60	0.5-1	94
Cent. America	7.57	24.55	7-8	31	Mexico	1.24	19.49	1-1.5	60
Cent. America	8.46	31.63	8-9	30	Mexico	1.77	31.61	1.5-2	103
Cent. America	9.62	29.77	9-10.21	32	Mexico	2.29	35.11	2-2.5	158
Central Andes	0.18	7.79	0-0.5	354	Mexico	2.74	32.18	2.5-3	182
Central Andes	0.73	17.78	0.5-1	141	Mexico	3.24	29.53	3-3.5	152
Central Andes	1.25	19.12	1-1.5	209	Mexico	3.80	29.77	3.5-4	186
Central Andes	1.78	25.41	1.5-2	276	Mexico	4.30	29.11	4-4.5	157
Central Andes	2.28	29.81	2-2.5	324	Mexico	4.73	25.98	4.5-5	141
Central Andes	2.73	32.51	2.5-3	270	Mexico	5.26	25.50	5-5.5	136
Central Andes	3.22	30.64	3-3.5	226	Mexico	5.72	29.20	5.5-6	129
Central Andes	3.73	28.71	3.5-4	133	Mexico	6.21	26.64	6-6.5	86
Central Andes	4.24	25.05	4-4.5	92	Mexico	6.73	22.31	6.5-7	90
Central Andes	4.72	23.72	5-5.5	75	Mexico	7.25	25.28	7-7.5	76
Central Andes	5.18	26.35	5-5.5	34	Mexico	7.80	22.82	7.5-8	62
Central Andes	5.80	25.07	5.5-6	27	Mexico	8.23	23.22	8-8.5	69
Central Andes	6.51	28.93	6-7	40	Mexico	8.75	24.81	8.5-9	55
Central Andes	7.30	28.29	7-7.5	29	Mexico	9.21	27.59	9-9.5	46
Central Andes	7.72	27.89	7.5-8	22	Mexico	9.76	26.77	9.5-10	22

Figure S1: Plots of Sr/Y versus MgO for the 9 arcs on thin (<20 km thick) crust.

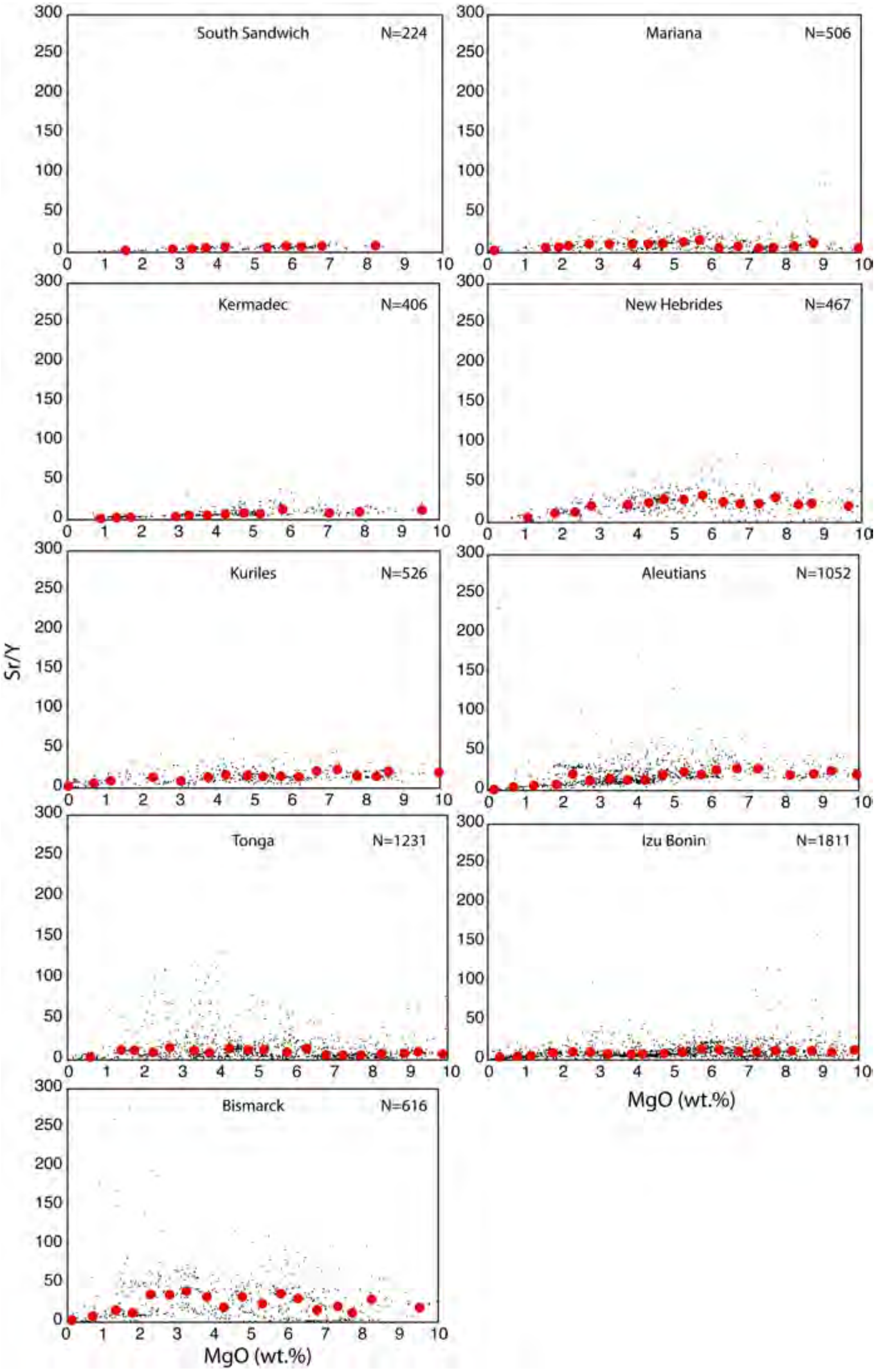

Small dots are individual analyses (N=number of analyses) from the Georoc database (http://georoc.mpch-mainz.gwdg.de/georoc/), whereas large red dots are median values calculated for intervals of ≥0.5 wt.% MgO (Tables S1-S2). Median values of Sr/Y comprised between ~2 and ~6 wt.% MgO were averaged for each one of the 22 arcs and corresponding 1σ uncertainties were calculated (error bars on Fig. 2). The averages of these median values are the dots of the plots of Figure 2.

Figure S2: Median values of Sr/Y versus MgO calculated from the dataset of each arc <20 km thick for MgO intervals ≥0.5 wt.% (see also Figure S4). Also shown are the exponential best fit curves to the points in each plot and the MgO (wt.%) value corresponding to the peak Sr/Y value estimated from the best fit curve. No peak values are shown for Mariana and Tonga because of the marked sinusoidal trends. For the meaning of the grey fields see footnote (d) of Table S1.

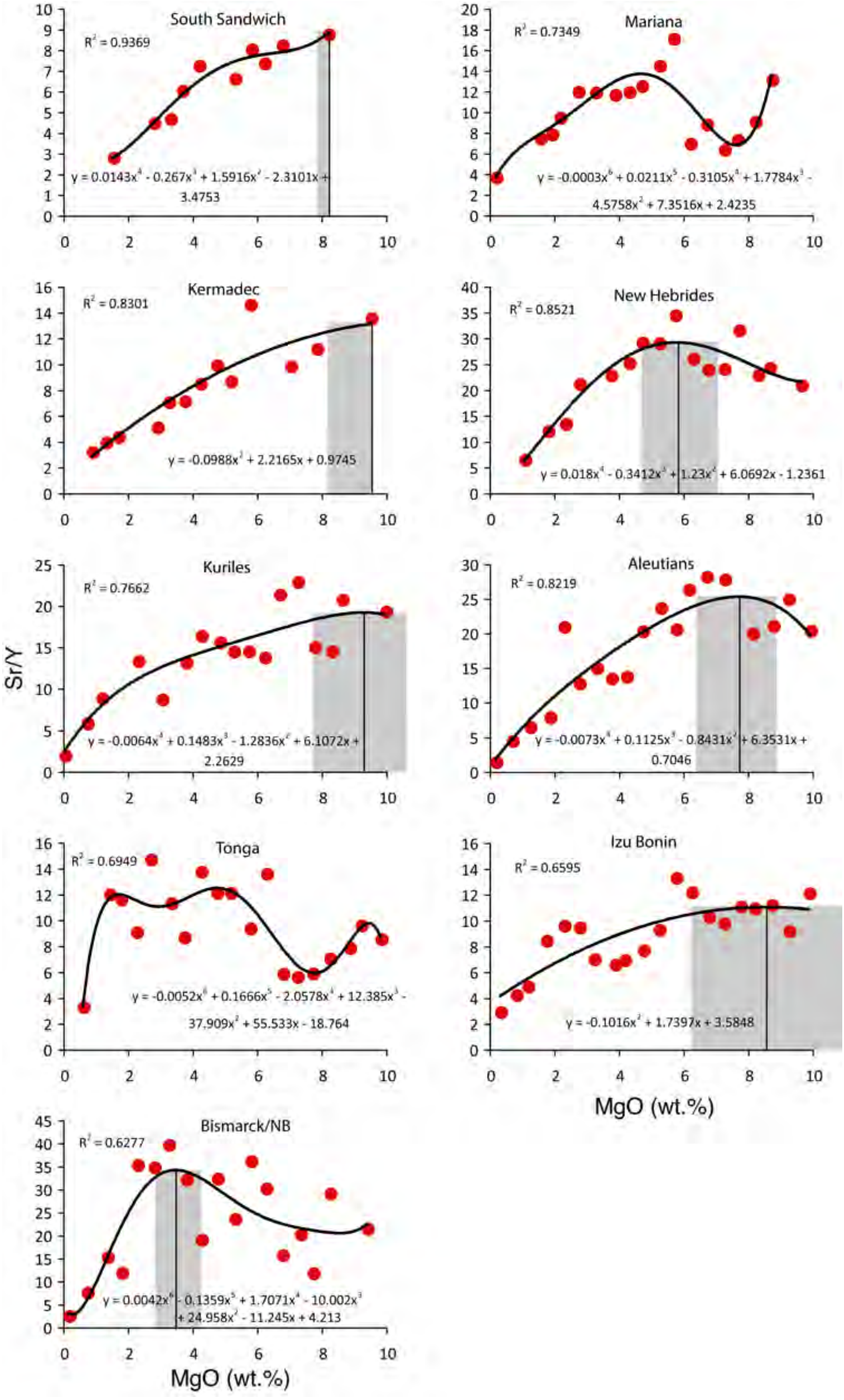

Figure S3: Plots of Sr/Y versus MgO for the 7 arcs on intermediate (20-30 km thick) crust.

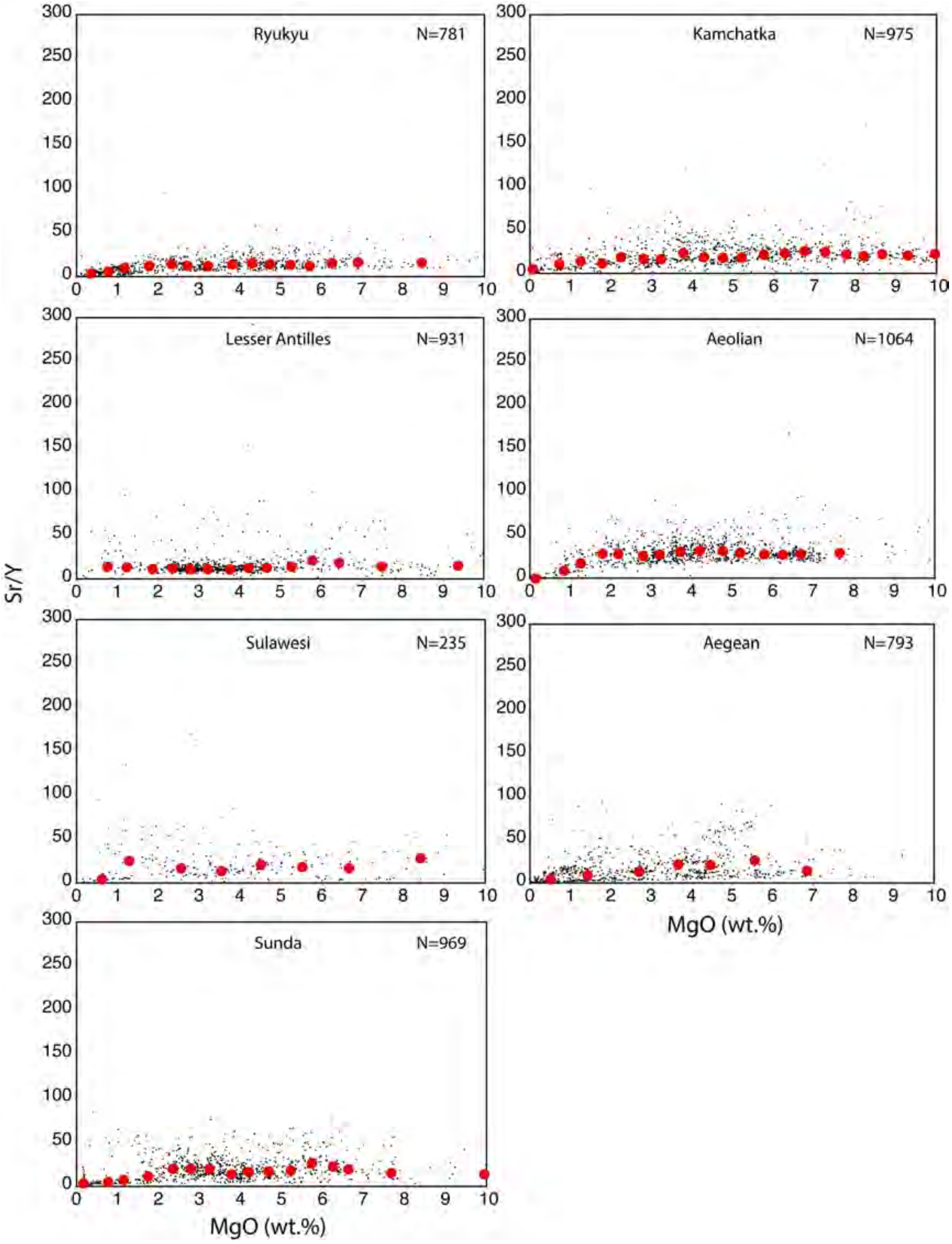

Small dots are individual analyses (N=number of analyses) from the Georoc database (http:// georoc.mpch-mainz.gwdg.de/georoc/Entry.html), whereas large red dots are median values calculated for intervals of ≥0.5 wt.% MgO (Tables S1-S2). Median values of Sr/Y comprised between ~2 and ~6 wt.% MgO were averaged for each one of the 22 arcs and corresponding 1σ uncertainties were calculated (error bars on Fig. 2). The averages of these median values are the dots of the plots of Figure 2.

Figure S4: Median values of Sr/Y versus MgO calculated from the dataset of each arc >20 and <30 km thick for MgO intervals ≥0.5 wt.% (see also Figure S5). Also shown are the exponential best fit curves to the points in each plot and the MgO (wt.%) value corresponding to the peak Sr/Y value estimated from the best fit curve. Note that no clear peak can be identified for Sulawesi arc due to the marked sinusoidal trend and the few data points. For the meaning of the grey fields see footnote (d) of Table S1.

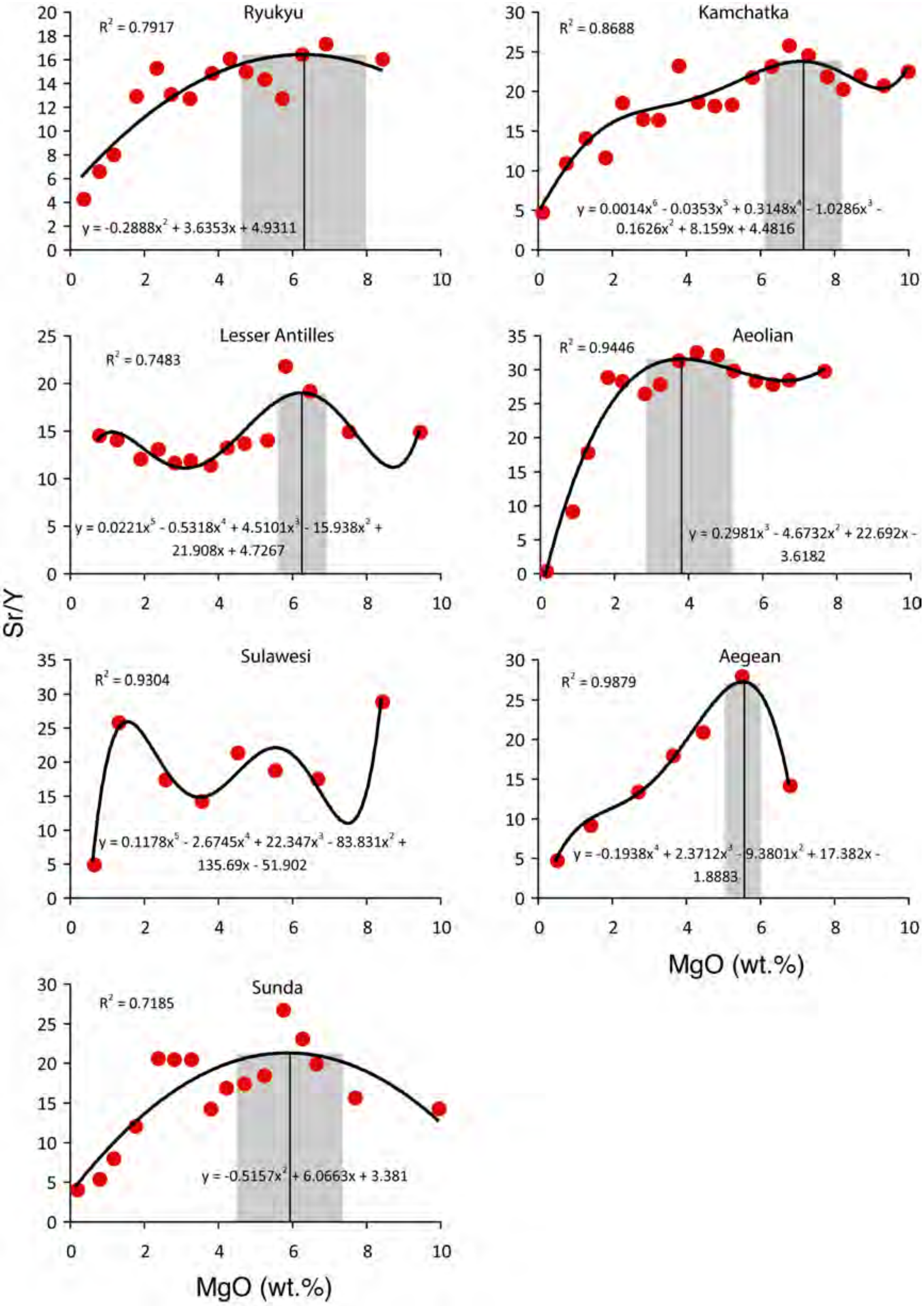

Figure S5: Plots of Sr/Y versus MgO for the 6 arcs on thick (>30 km thick) crust.

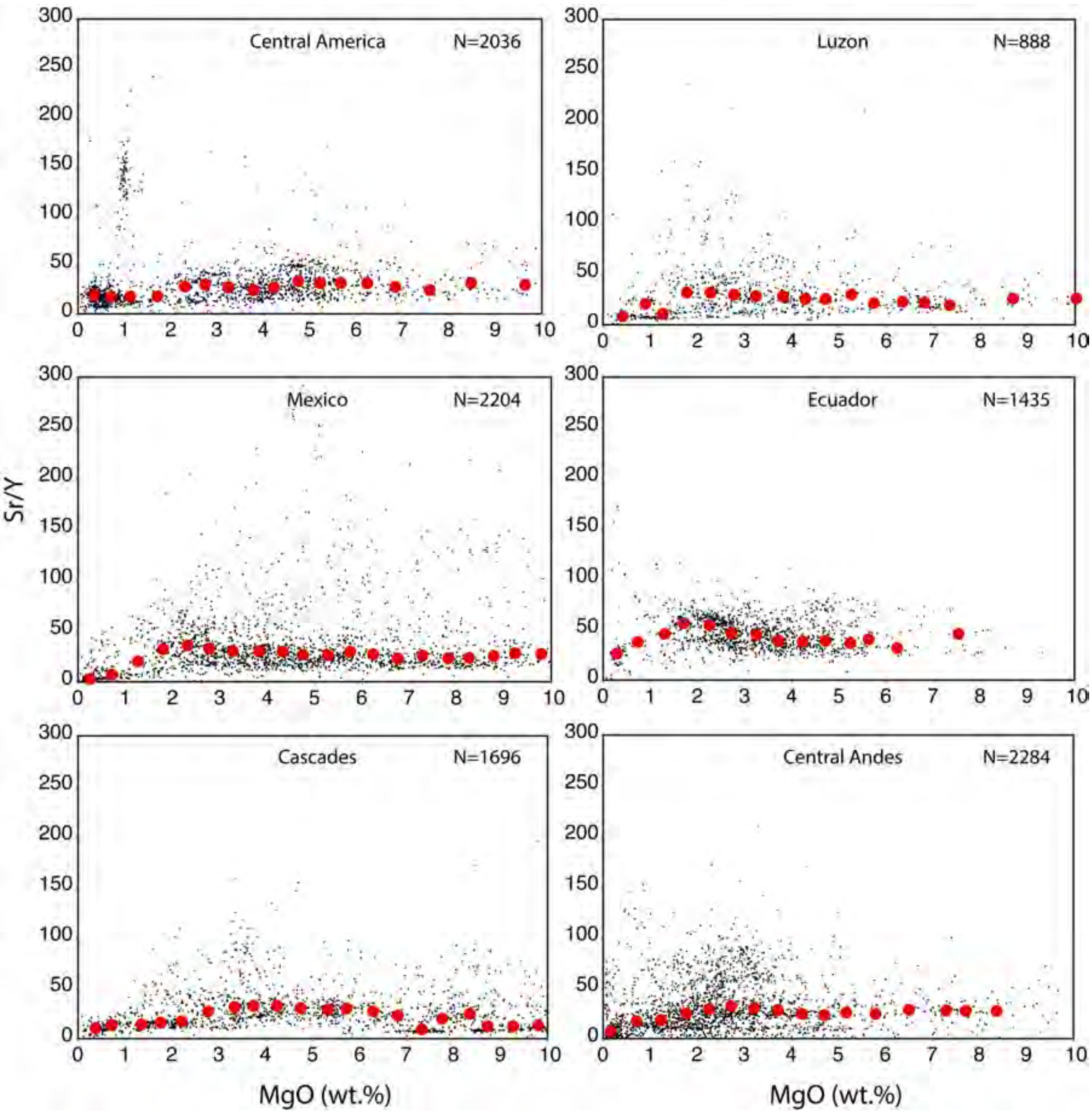

Small dots are individual analyses (N=number of analyses) from the Georoc database (http://georoc.mpch-mainz.gwdg.de/georoc/), whereas large red dots are median values calculated for intervals of ≥0.5 wt.% MgO (Tables S1-S2). Median values of Sr/Y comprised between ~2 and ~6 wt.% MgO were averaged for each one of the 22 arcs and corresponding 1σ uncertainties were calculated (error bars on Fig. 2). The averages of these median values are the dots of the plots of Figure 2.

The cluster of high Sr/Y values at around 1 wt.% MgO for Central America is from El Valle volcano, interpreted to derive from high-pressure fractionation of garnet from hydrous magmas (Hidalgo et al., 2011).

Reference

Hidalgo, P.J. *et al.* Origin of silicic volcanism in the Panamanian arc: evidence for a two-stage fractionation process at El Valle volcano. *Contrib. Min. Pet.* **162**, 1115-1138 (2011).

Figure S6: Median values of Sr/Y versus MgO calculated from the dataset of each arc >30 km thick for MgO intervals ≥0.5 wt.% (see also Figure S6). Also shown are the exponential best fit curves to the points in each plot and the MgO (wt.%) value corresponding to the peak Sr/Y value estimated from the best fit curve. For the meaning of the grey fields see footnote (d) of Table S1.

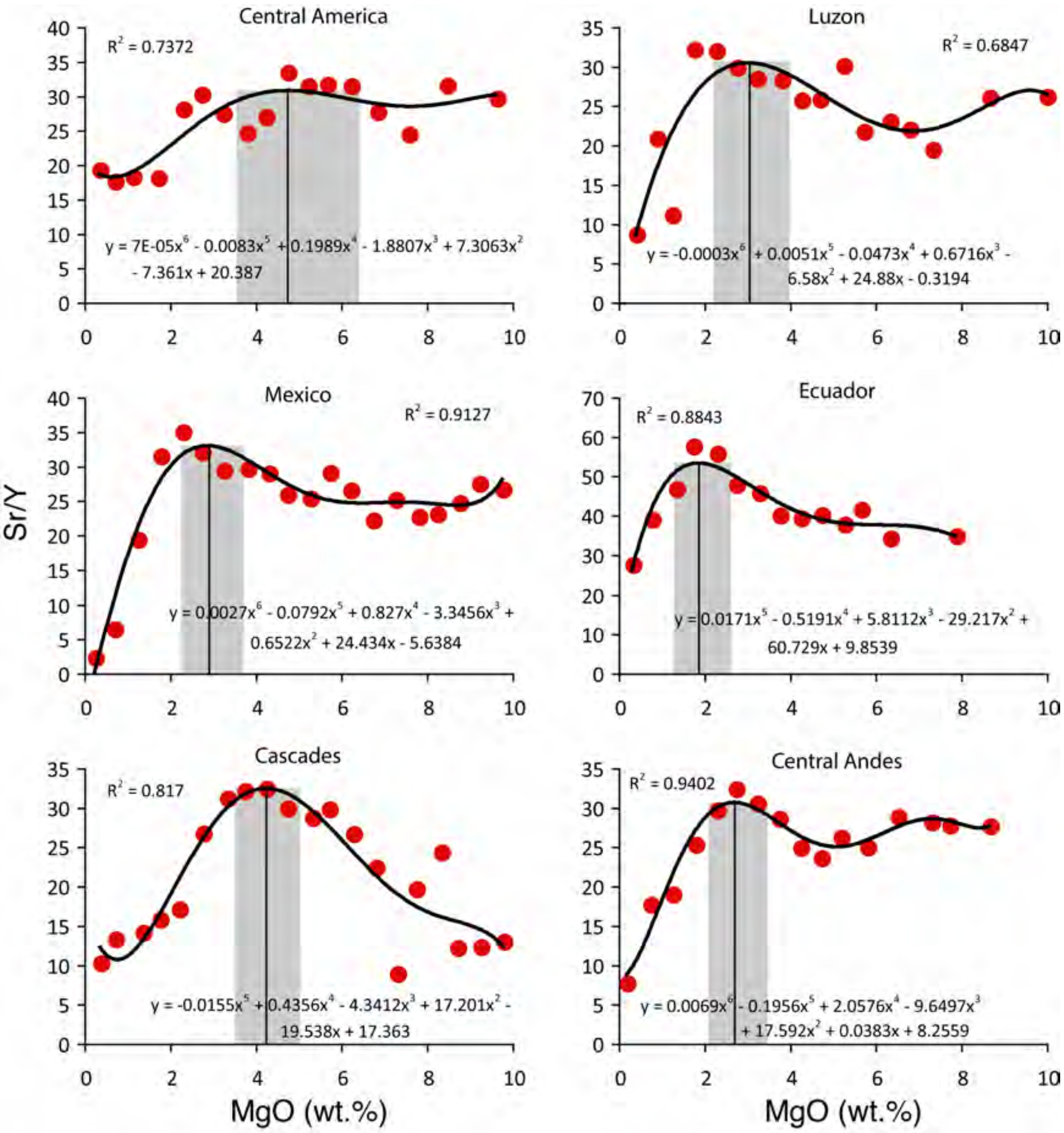

Table S5: Compositions of parent magmas and fractionating assemblages for fractional crystallization modeling in Figure 3. Concentrations of all oxides are in wt.%, those of Sr and Y in ppm.

	arcs >30 km	arcs 20-30 km (10→5.5 MgO)	arcs 20-30 km (5.5-0 MgO)	arcs <20km
	continental arc basalt [1]	oceanic arc basalt [1]	Kamchatka arc andesite[2]	average oceanic arc[1]
SiO_2	51.33	50.46	53	50.46
TiO_2	0.98	0.91	1.36	0.91
Al_2O_3	15.7	15.72	17.5	15.72
FeO	8.72	8.52	8.27	8.52
MnO	0.17	0.17	0.16	0.17
MgO	9.48	9.84	5.53	9.84
CaO	9.93	11.44	7.59	11.44
Na_2O	2.61	2.35	4.11	2.35
K_2O	0.88	0.45	1.64	0.45
P_2O_5	0.22	0.15	0.61	0.15
Sr[a]	400	380	438	280
Y[a]	20	20	20	20
Total	100.02	100.01	99.77	100.01
Fractionating assemblage (mineral fraction)				
olivine	0.2	0.3	0	0.5
clinopyroxene	0.55	0.55	0.72	0.25
amphibole	0.25	0.15	0	0
plagioclase	0	0	0.28	0.25
Total	1	1	1	1

[a]values adjusted to fit with the least evolved point of the average trend

References

[1] Kelemen, P.B., Hanghoj, K. & Greene, A.R. One view of the geochemistry of subduction-related magmatic arcs, with an emphasis on primitive andesite and lower crust. In: Rudnick, R.L. (Ed.), *The Crust*, Elsevier, Amsterdam, pp. 593-659 (2004).

[2] Sample AB0262 in Volynets, A.O. *et al.* Mafic late Miocene-Quaternary volcanic rocks in the Kamchatka back-arc region: implications for subduction geometry and slab history at the Pacific-Aleutian junction. *Contrib. Min. Pet.* **159**, 659-687 (2010).

Table S6: Compositions of fractionating minerals (Table S4) for fractional crystallization modeling in Figure 3. Concentrations of all oxides are in wt.%, those of Sr and Y in ppm.

Mineral<br>Sample	olivine<br>E05024bis_11_ol1[1]	amphibole<br>E05150_3_4[2]	plagioclase<br>pl67_1-2[2]	clinopyroxene<br>E05022_9_cpx1_7[1]
SiO_2	39.98	44.25	47.38	53.29
TiO_2	0.02	1.30	0.00	0.25
Al_2O_3	0.02	11.77	33.60	2.29
Cr_2O_3	0.03	0.01	0.00	0.44
FeO	12.57	11.28	0.62	4.55
MnO	0.22	0.15	0.00	0.10
MgO	47.17	15.61	0.03	17.11
CaO	0.10	11.14	16.44	22.13
Na_2O	0.00	2.04	1.80	0.31
NiO	0.25	0.02	0.00	0.00
K_2O	0.00	0.56	0.09	0.00
H_2O	0.00	1.64	0.00	0.00
Sr	0	20	1500	20
Y	0	50	0	10
Total	100.35	99.74	99.96	100.46

References

[1] Chiaradia, M., Müntener, O. & Beate, B. Quaternary Sanukitoid-like Andesites Generated by Intracrustal Processes (Chacana Caldera Complex, Ecuador): Implications for Archean Sanukitoids. *J. Pet.* **55**, 769-802 (2014).

[2] Chiaradia, M., Müntener, O. & Beate, B. Enriched Basaltic Andesites from Mid-crustal Fractional Crystallization, Recharge, and Assimilation (Pilavo Volcano, Western Cordillera of Ecuador). *J. Pet.* **52**, 1107-1141 (2011).

Figure S7: Averages of median Sr/Y values of the arc groups subdivided by thickness intervals and of Archean greenstone belt rocks (Baltic shield, Western Australia, Superior Province and Tanzania) versus MgO. Also shown are modeled fractional crystallization trends reproducing the three arc groups (tick marks and numbers represent percent of remaining melt). For parameters used in the modeling see Tables S5-S6.

The average trend of Sr/Y versus MgO in thick arcs is reproduced by fractionation of amphibole-clinopyroxene-olivine and no plagioclase down to ~2 wt.% MgO (corresponding to ~37% fractionation of solid phases): this is atypical high-pressure fractionation assemblage. The average trend in thin arcs is reproduced by continuous fractionation of olivine-pyroxene-plagioclase down to ~2 wt.% MgO (corresponding to ~30% fractionation of these mineral phases): this is a typical low pressure fractionating assemblage. The average trend in intermediate arcs is reproduced by a two-step fractionation process: an early one characterized by olivine-clinopyroxene-amphibole (down to ~5.5 wt.% MgO corresponding to 20% fractionation) and a second one by olivine-pyroxene-plagioclase (down to ~2 wt.% MgO corresponding to 40% fractionation): this evolution is typical of a mixed high and low-pressure environment.

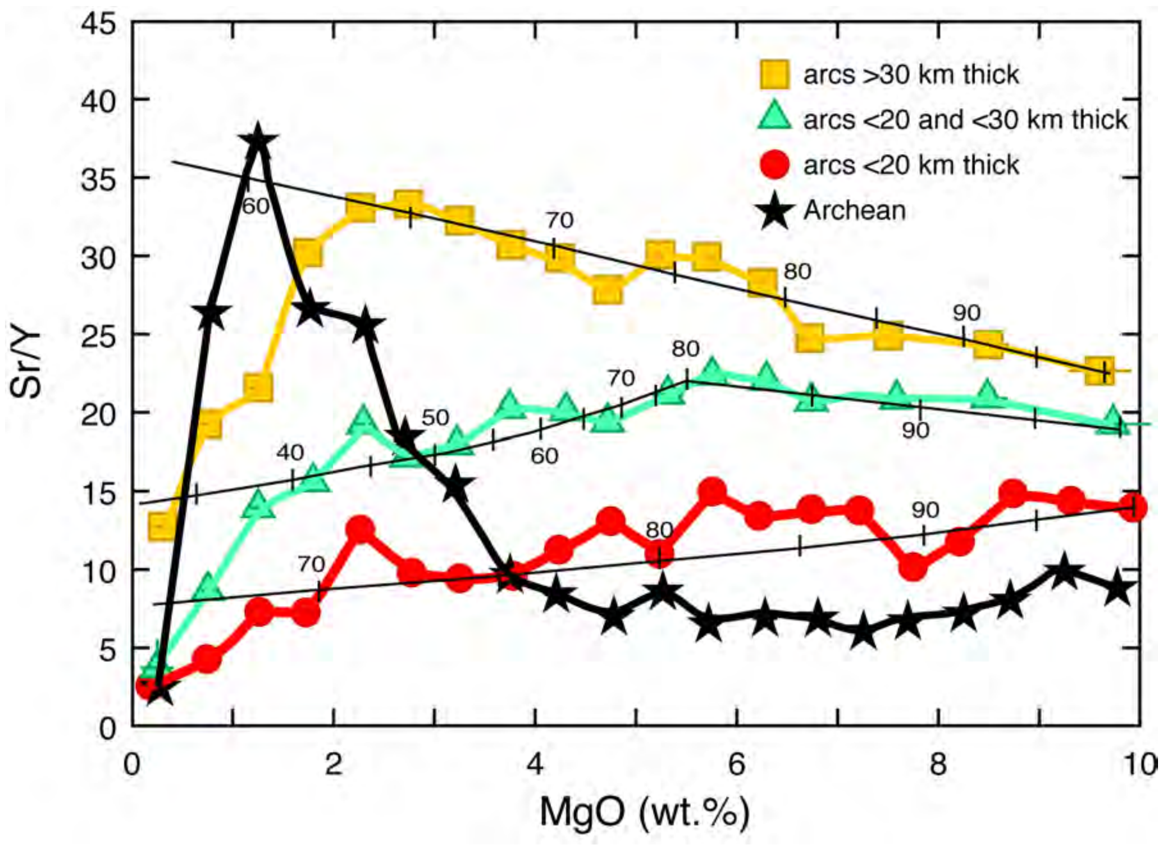

Table S7: Summary of median values of MgO and Sr/Y for Archean greenstone belts calculated for the MgO intervals indicated. N = number of points within each interval.

Province	MgO	Sr/Y	Interval	N	Province	MgO	Sr/Y	Interval	N
Baltic Shield	0.34	3.6	0-0.5	24	W. Australia	0.28	1.8	0-0.5	35
Baltic Shield	0.72	39.3	0.5-1	53	W. Australia	0.81	5.4	0.5-1	28
Baltic Shield	1.26	30.0	1-1.5	61	W. Australia	1.13	57.9	1-1.5	35
Baltic Shield	1.74	31.4	1.5-2	43	W. Australia	1.80	30.6	1.5-2	19
Baltic Shield	2.25	36.3	2-2.5	59	W. Australia	2.32	10	2-2.5	32
Baltic Shield	2.69	30.2	2.5-3	35	W. Australia	2.72	7.7	2.5-3	29
Baltic Shield	3.61	12.8	3-4	42	W. Australia	3.23	6.7	3-3.5	31
Baltic Shield	4.20	9.4	4-4.5	32	W. Australia	3.72	6.3	3.5-4	28
Baltic Shield	4.78	8.5	4.5-5	32	W. Australia	4.28	6.7	4-4.5	46
Baltic Shield	5.28	7.4	5-5.5	30	W. Australia	4.74	4.8	4.5-5	45
Baltic Shield	5.74	7.6	5.5-6	47	W. Australia	5.26	8.8	5-5.5	51
Baltic Shield	6.23	7.9	6-6.5	54	W. Australia	5.78	5.7	5.5-6	61
Baltic Shield	6.83	7.4	6.5-7	67	W. Australia	6.16	5.2	6-6.5	71
Baltic Shield	7.26	6.0	7-7.5	68	W. Australia	6.76	6.7	6.5-7	66
Baltic Shield	7.75	6.9	7-7.5	77	W. Australia	7.23	5.7	7-7.5	57
Baltic Shield	8.26	6.4	8-8.5	72	W. Australia	7.75	6.6	7.5-8	63
Baltic Shield	8.73	6.3	8.5-9	60	W. Australia	8.22	6.9	8-8.5	43
Baltic Shield	9.19	6.5	9-9.5	41	W. Australia	8.66	8.4	8.5-9	35
Baltic Shield	9.96	6.4	9-5-10.4	43	W. Australia	9.61	9.2	9-10	42
Superior	0.32	3.0	0-0.5	124					
Superior	0.77	4.7	0.5-1	147					
Superior	1.24	20.8	1-1.5	90					
Superior	1.76	17.7	1.5-2	83					
Superior	2.24	13.5	2-2.5	75					
Superior	2.72	17.4	2.5-3	62					
Superior	3.23	13.5	3-3.5	72					
Superior	3.77	10.8	3.5-4	78					
Superior	4.31	8.9	4-4.5	77					
Superior	4.80	8.7	4.5-5	86					
Superior	5.26	9.5	5-5.5	80					
Superior	5.77	6.1	5.5-6	95					
Superior	6.30	8.2	6-6.5	89					
Superior	6.83	6.5	6.5-7	74					
Superior	7.28	6.4	7-7.5	75					
Superior	7.71	8.5	7.5-8	63					
Superior	8.27	8.2	8-8.5	65					
Superior	8.70	10.7	8.5-9	43					
Superior	9.29	11.5	9-9.5	39					
Superior	9.80	11.8	9.5-10.1	43					
Tanzania	0.18	1.0	0-0.5	28					
Tanzania	0.86	45.2	0.5-1.5	21					
Tanzania	2.40	40.7	1.5-3	24					
Tanzania	4.02	8.6	3-4.5	24					
Tanzania	4.84	5.9	4.5-5	24					
Tanzania	5.59	6.7	5-6	29					
Tanzania	6.41	6.2	6-7	29					
Tanzania	7.94	6.2	7-9.5	18					

Figure S8: Plots of Sr/Y versus MgO for 4 Archean greenstone belt provinces.

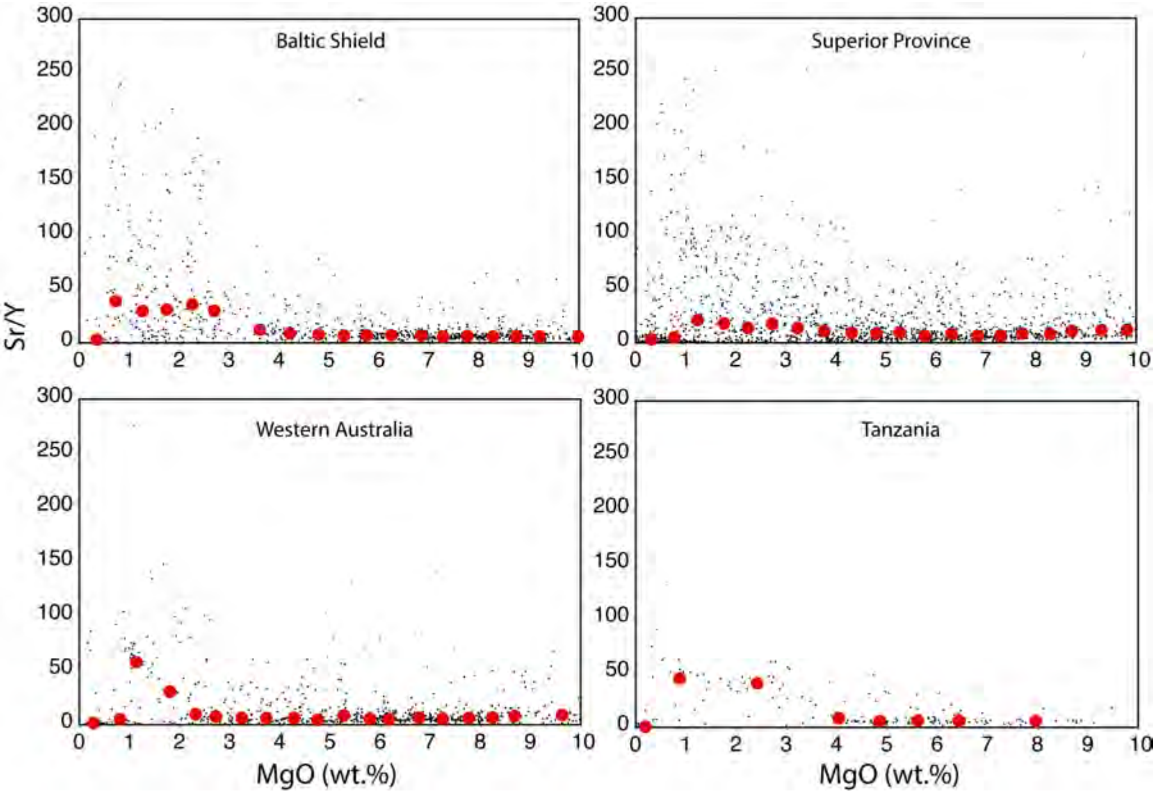

Small dots are individual analyses (N=number of analyses) from the Georoc database (http://georoc.mpch-mainz.gwdg.de/georoc/), whereas large red dots are median values calculated for intervals of ≥0.5 wt.% MgO (Tables S1-S2). Median values of Sr/Y comprised between ~2 and ~6 wt.% MgO were averaged for each one of the 22 arcs and corresponding 1σ uncertainties were calculated (error bars on Fig. 2). The averages of these median values are the dots of the plots of Figure 2.
